# A case of peritoneal dialysis-associated peritonitis caused by *Rhodococcus kroppenstedtii*

**DOI:** 10.1186/s12879-021-06280-8

**Published:** 2021-06-13

**Authors:** Yi Kang, Yuxin Chen, Zhifeng Zhang, Han Shen, Wanqing Zhou, Chao Wu

**Affiliations:** 1 Department of Infectious Diseases, Nanjing Drum Tower Hospital, Clinical College of Nanjing Medical University, 321# Zhongshan Road, Gulou District, Nanjing City, Jiangsu Province 210008 P. R. China; 2 Department of Laboratory Medicine, Nanjing Drum Tower Hospital, the Affiliated Hospital of Nanjing University Medical School, Nanjing, Jiangsu Province 210008 P. R. China

**Keywords:** *Rhodococcus kroppenstedtii*, Peritoneal dialysis-associated peritonitis, Whole genome sequencing, Average nucleotide identity

## Abstract

**Background:**

*Rhodococcus kroppenstedtii* is an aerobic, gram-positive bacterium firstly identified in the environment, which has not been reported in human-related infection. Herein, we reported the first case of peritoneal dialysis (PD)-associated peritonitis caused by *R. kroppenstedtii* which was identified by whole genome sequencing.

**Case presentation:**

A 69-year-old man was admitted to hospital with abdominal pain and fever. Over the last 2 years, he had been undergoing continuous ambulatory peritoneal dialysis (CAPD) due to end-stage renal disease. Clinical symptom and sign in combination with laboratory examinations supported the clinical diagnosis of PD-associated peritonitis. Thus, ceftizoxime and teicoplanin were empirically used after PD effluent was collected for bacterial culture. A gram-positive bacterium was found from the PD effluent culture, which could not be identified by either Vitek 2 Compact ANC card or matrix-assisted laser desorption/ionization time-of-flight mass spectrometry. The strain was finally confirmed to be *R. kroppenstedtii* by whole genome sequencing (WGS) through the average nucleotide identity (ANI) analysis. With a continuous treatment with teicoplanin and imipenem for 15 days and intraperitoneal catheter removed, the infection symptom was improved evidenced by a normal body temperature, also with white blood cell count (WBC), procalcitonin (PCT) and C-reactive protein (CRP) dropped to normal levels. Peritoneal dialysis effluent culture showed a negative result. Then, hemodialysis and arteriovenous fistula angioplasty were performed, but the patient developed a progressive blood pressure loss, accompanied by multiple organ disorder, and died on Feb 25, 2020.

**Conclusions:**

To the best of our knowledge, this is the first time to report a peritoneal dialysis-associated peritonitis caused by *R. kroppenstedtii* which was identified by average nucleotide identity analysis based on WGS.

## Background

*Rhodococci* are aerobic, gram-positive, partially acid-fast, catalase-positive, non-motile, non-spore-forming, rod-and coccoid-like bacteria which can be isolated from various sources of the environment and usually infect immunocompromised patients such as Acquired Immune Deficiency Syndrome (AIDS) or transplant recipients [1, 2]. In the past decades, due to the advantage of improved taxonomic methodology, including both chemotaxonomic and molecular based approaches, many species have been established or reclassified and 73 named species (https://lpsn.dsmz.de/genus/rhodococcus) are included in *Rhodococci* genus. *R. equi* is the most described human pathogen which causes pneumonia in immunocompromised humans [1–3], while few case reports about *Rhodococcus* spp. causing peritonitis were reported [4, 5]. Extrapulmonary infection was reported including brain abscess, complicated skin and soft tissue infection, bloodstream infection, lymphadenitis, and pericarditis [1, 2]. To the best of our knowledge, *Rhodococcus kroppenstedtii*, which was firstly isolated in the cold desert of Himalayas, India, has not been previously described as a human pathogen [6]. Herein, we present the first case of peritoneal dialysis (PD)-associated peritonitis caused by *R. kroppenstedtii* which was identified by whole genome sequencing (WGS) and average nucleotide identity (ANI) analysis in China.

## Case presentation

A 69-year-old man was admitted to our hospital on Jan 25, 2020 with a complaint of fever and abdominal pain for 1 days’ duration. The patient was diagnosed with hypertension and cerebral infarction 10 years ago, and also with a medical history of renal anemia and cholecystectomy. In 2014, the patient was diagnosed with IgA nephropathy by a renal biopsy and was treated with diet. In 2018, the patient progressed to chronic kidney disease stage 5 and underwent peritoneal dialysis for 2 years until this admission.

    At the emergency department on Jan 25, 2020, his physical examination showed a body temperature of 38.6 °C, a blood pressure of 206/95 mmHg, a pulse rate of 85 beats/min, and a respiratory rate of 18 breaths/min. He complained tenderness and rebound pain in his abdomen, with pitting lower extremity edema. Redness or discharge was not found near the peritoneal catheter. Laboratory tests revealed a white blood cell count (WBC) of 13.9 × 10^9^/L with 80.7% neutrophils, a hemoglobin (Hb) level of 74 g/L, a C-reactive protein (CRP) level of 96.7 mg/L, a procalcitonin (PCT) level of 3 ng/ml, a B-type natriuretic peptide (BNP) level of 939 pg/mL, an urea level of 25.27 mmol/L and a creatinine level of 924.2 μmol/L. Routine testing of the PD effluent revealed a yellow and muddy appearance with positive results in the Rivalta test, WBC count of 13,363 × 10^6^/L (neutrophils: 98%). Peritoneal dialysis-associated peritonitis was diagnosed initially. The PD effluent was collected for bacterial culture.

    Being admitted to the Nephrology ward later in the same day, another PD effluent was collected for bacterial culture before starting an empirical antibiotic therapy. Then, the patient was empirically treated with ceftizoxime intravenously (2 g bid iv) and intraperitoneal injection of teicoplanin (0.2 g qd ip) for 7 days. On Feb 1, a gram-positive bacterium was found from PD effluent culture. Therefore, teicoplanin (0.2 g qd iv) and imipenem (0.5 g qd ip) were administered based on the antimicrobial susceptibility testing for the next 4 days. Due to the low blood concentration of teicoplanin and intraperitoneal catheter removed on Feb 5, the treatment was adjusted to teicoplanin (0.4 g qd iv) and imipenem (0.5 g q12h iv). With the continuous treatment with teicoplanin and imipenem for 15 days, the infection symptom was improved with a normal body temperature of 36.6 °C, a PCT level of  0.7 ng/ml and a CRP level of  76.8 mg/L. Routine testing of the peritoneal fluid were within normal ranges. Peritoneal dialysis effluent culture and intraperitoneal catheter culture also showed negative result.

    Thereafter, arteriovenous fistula angioplasty was performed for hemodialysis on Feb 15 and the patient developed fever (38 °C) and disturbance of consciousness on the first day after surgery. No cerebral hemorrhage and cerebral infarction were found by cranial Computed Tomography (CT). Considered the toxicity, imipenem was switched to biapenem. However, the patient’s symptoms and signs did not improve, and the laboratory test indicators gradually increased. On Feb 17, elevated PCT (0.801 ng/mL) and CRP (76.8 mg/L) was observed. Blood culture showed a negative result, and amikacin was given instead of biapenem for the treatment. On Feb 20, the patient still had a fever (37.8 °C) and thick sputum. Laboratory examination revealed a PCT of 1.34 ng/mL, a CRP of 58.5 mg/L, a ESR of more than 140 mm/h, a Hb of 45 g/L. The fecal occult blood test was positive and the blood culture was negative. High resolution CT of the thorax showed inflammatory manifestations including pulmonary cord, flocculent shadow and exudate. Unfortunately, the patient died on Feb 25 due to septic shock and multi-organ failure.

## **Bacteria culture and identification**

On Jan 25, 2020, twenty milliliters of PD effluent were drawn and injected into aerobic and anaerobic blood culture bottles (bioMérieux, France), and sent to the Bacter/Alter 3D automated blood culture system (bioMérieux, France). At the same time, another ten milliliters of PD effluent were centrifuged for smear with gram-positive bacilli upon gram staining (Baso, Zhuhai, China). After culturing for 55 h, a positive signal was observed in the aerobic blood culture bottle, and the culture fluid was directly smeared with gram-positive bacilli which was consistent to the directly smear result before. No bacteria were found in anaerobic medium with 120 h of culture. After his admission, another ten milliliters of PD effluent were collected into aerobic blood culture bottle on Jan 25 with a positive culture result after 54 h. Positive culture fluid was transferred to Columbia blood agar (OXOID) and incubated at 35 °C 5% CO_2_. A few small colonies were observed after 24 h of incubation; 48 h later, orange-red colonies were observed; with another 24 h culture, dry, rough, convex and orange-red-pigmented was observed. Catalase was produced; acid-fast and weakly acid-fast staining were negative. The strain we obtained was named JXM12926.

**Fig. 1:**
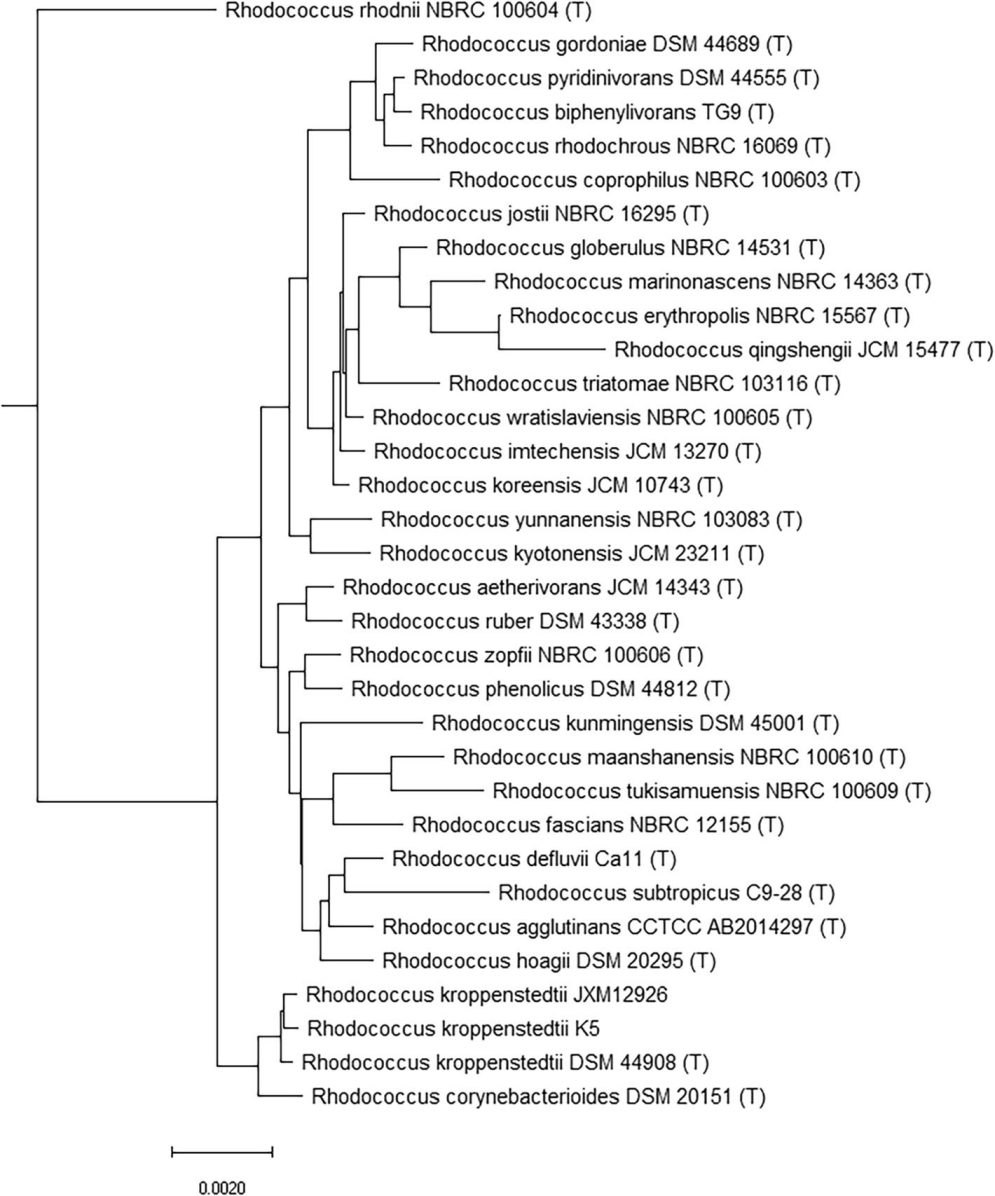
The phylogenetic tree of *Rhodococcus* spp. based on the WGS was constructed by neighbor-joining method and kimura 2-parameter model with using the MEGA 6 software. The scale bar shows the genetic distance. The number presented next to each node shows the percentage bootstrap value of 1000 replicates

    Biochemical identification of JXM12926 was carried out by using Vitek 2 Compact ANC card (bioMérieux, France) according to the manufacturer’ instructions which identified to *Acinomyces odontolyticus* and *Turicella otitidis* with 50% identification rate respectively. Additional testing using the VITEK mass spectrometry v3.0 (bioMérieux, France) failed to identify the strain. Genomic DNA of JXM12926 was prepared using the QIAamp DNA mini kit (Qiagen, Hilden, Germany) and was subjected to whole-genome sequencing using the Ion Torrent Personal Genome Machine (Life Technologies, USA). Library construction, sequencing reactions were performed according to the manufacturer’s instructions and a 300 pair-end library was generated. The resulting sequences were de novo assembled using SOAPdenovo v2.01 (http://soap.genomics.org.cn/, v2.01) [7]. For the prokaryotic organism, we used ab initio prediction method to get gene models for strain JXM12926. Gene models were identified using Glimmer 3 [8]. All gene models were then blasted against non-redundant (NR in NCBI) database, SwissProt (http://uniprot.org), KEGG (http://www.genome.jp/kegg/) [9], and COG (http://www.ncbi.nlm.nih.gov/COG) [10], to do functional annotation. The ANI between JXM12926 and its phylogenetic neighbors was analyzed by the software Orthologous Average Nucleotide Identity Tool (OAT) (http://www.ezbiocloud.net/tools/orthoani), using ncbi-blast-2.2.30+ search under the Java Runtime Environment Version 8 [11]. The chromosome comprises 3, 977, 933 bp with a G + C content of 70.1% which was similar with *R. kroppenstedtii* DSM 44908. A total of 86 contigs was generated and the N50 contig size was 110, 332 bp. By using complete genome sequence, we found a 98.05% ANI between strain JXM12926 and *R. kroppenstedtii* DSM 44908*.* The phylogenetic tree was constructed using the neighbor-joining and Tamura-Nei methods [12] in the software package MEGA 6 (default settings) which was showed in Fig. 1*.* This whole Genome Shotgun project has been deposited at DDBJ/ENA/GenBank under the accession JACEFU000000000. The version described in this study is version JACEFU010000000.

## **Antimicrobialsusceptibility testing**

Antimicrobial susceptibility testing (AST) was performed using the E-test method on MH agar (OXOID) including penicillin, ceftriaxone, ceftazidime, imipenem, erythromycin, clindamycin, levofloxacin, vancomycin, and linezolid (Autobio, China) and K-B method including amikacin, tetracycline and sulfamethoxazole (OXOID). The *Staphylococcus aureus* ATCC29213 and ATCC25923 was included as quality controls. After 48 h of incubation at 35 °C, strain JXM12926 showed high minimum inhibitory concentration (MIC) value to ceftazidime (> 256 μg/mL) while low MICs to penicillin, imipenem, erythromycin, levofloxacin, as showed in Table 1.

**Table 1:** Antimicrobial susceptibility testing of *R. kroppenstedtii* strain JXM12926

Antibiotics	MIC (μg/mL)	K-B (mm)
Penicillin	0.5	
Ceftriaxone	8	
Ceftazidime	> 256	
Imipenem	0.125	
Erythromycin	0.25	
Clindamycin	2	
Levofloxacin	0.048	
Vancomycin	0.5	
Linezolid	1	
Amikacin		32
Tetracycline		28
Sulfamethoxazole		6

## Discussion and conclusions

*Rhodococcus* species are generally considered as opportunity human pathogens, in which *R. equi* is the most described cause of pheumonia in immunocompromised hosts such as AIDS and transplant recipients [1, 3]. Besides, other *Rhodococcus* human pathogens including *R. bronchialis* [13, 14], *R. rhodochrous* [15], *R. rubropertinctus* [16], *R. aurantiacus* [17], *R. rhodnii* [17], *R. corynebacterioides* [18], and *R. fascians* [19] are also reported.

    Peritonitis is the main infectious complication in patients undergoing continuous ambulatory peritoneal dialysis (CAPD) in which gram-positive bacteria (60–80% of all isolates) are the most common pathogens [20]. Bacteria from the skin flora and environmental microbes entering the peritoneal cavity through the catheter is the most common cause of infection [20]. There are few reports of PD-associated peritonitis caused by *Rhodocuccus* spp., including *R. equi, R. erythropolis* and *R. bronchialis* [4, 5, 21–23]. We report the first clinical PD-associated peritonitis caused by *R. kroppenstedtii* which was identified by WGS and ANI. *R. kroppenstedtii* was firstly isolated in the cold desert of the Himalayas, India and established in 2006 [6], while no human infectious report as we known.

*    R. kroppenstedtii* could not be identified by Vitek 2 Compact ANC and matrix-assisted laser desorption/ionization time-of-flight mass spectrometry. Among nucleic acid-based methods, the most powerful approach for revealing reliable phylogenetic relationships is 16S rRNA gene sequence analysis, but it failed to differentiate some *Rhodococcus* species [2]. In 2019, a case of neonatal oligoarthritis and bactermia caused by *Rhodococcus* which could not be differentiated between *R. corynebacterioides* and *R. kroppenstedtii* by 16S rRNA sequence [18], while DNA-DNA hybridization methods is recommended for the delineation of such species [2].

    Accurate species-level identification of isolates from clinical specimens is crucial for understanding their pathogenesis. Unfortunately, most commercial available detection systems are not capable for the identification of *Rhodococcus* species, suggesting a potential pitfall in conventional clinical microbiology laboratory. Whole genome sequencing provides the opportunity of a comprehensive understanding for genome of bacteria, which has been widely used for bacterial classification, evolution, and surveillance of antibiotic resistant genes [2]. The average pairwise genome aggregate ANI between genomes with JXM12926 and *R. kroppenstedtii* DSM 44908 was 98.05%, up the 95% ANI cutoff frequently used for species demarcation [11], and supporting the classification of JXM12926 into *R. kroppenstedtii.* The phylogenetic tree constructed by the OrthoANI distance of the whole genome sequence showed that JXM12926 was grouped into the *R. kroppenstedtii* cluster.

    Empirical treatment for peritonitis are anti-positive bacteria according International Society for Peritoneal Dialysis (ISPD) guideline [20], while no recommendations for treatment of *Rhodococcus*-induced peritonitis is included in the guideline. It is reported that macrolide family together with rifampicin are the most effective antibiotic agents for the treatment of *Rhodococcus* [24]. *R. kroppenstedtii* JXM12926 in our study shown susceptible to carbapenems, glycopeptides and penicillin. It is suggested that a combination of at least two antimicrobial agents should be used for treatment, including an antibiotic with intracellular activity [2]. Our patient initially received intravenous ceftizoxime, intraperitoneal injection of teicoplanin for empirical treatment. Given the drug sensitivity results, the treatment was changed to intravenous teicoplanin and imipenem. Then, the patient rapidly improved. However, after the arteriovenous fistula angioplasty and discontinuation of imipenem, the patient’s infection symptoms reappeared. It was uncertain whether the recurrence of infection is related to the previous infection control.

    In conclusion, to our knowledge, this is the first case of peritoneal dialysis-associated peritonitis caused by *R. kroppenstedtii* which was identified by average nucleotide identity analysis based on WGS. The identification process for this strain JXM12926 is sufficient to provide a limited data support for the detailed study on this species of *R. kroppenstedtii*.

## Data Availability

The datasets used and/or analyzed during the current study are available from the corresponding author on reasonable request. The genome of JXM12926 is available in the DDBJ/ENA/GenBank repository, under the accession JACEFU000000000.

## Abbreviations

PD: Peritoneal Dialysis
CAPD: Continuous Ambulatory Peritoneal Dialysis
WGS: Whole genome sequencing
ANI: Average nucleotide identity
WBC: White blood cell
PCT: Procalcitonin
CRP: C-reactive protein
AIDS: Acquired Immune Deficiency Syndrome
Hb: Hemoglobin
BNP: B-type natriuretic peptide
CT: Computed tomography
AST: Antimicrobial susceptibility testing
MIC: Minimum Inhibitory Concentration
ISPD: International Society for Peritoneal Dialysis
